# Resilience changes and occupational resilience factors among healthcare workers during and after the COVID-19 pandemic: A 2-year prospective cohort study

**DOI:** 10.1038/s41598-025-09829-8

**Published:** 2025-08-10

**Authors:** Papoula Petri-Romão, Gonzalo Martínez-Alés, Irene Martinez-Morata, Berta Moreno-Küstner, Eduardo Fernández-Jiménez, Irwin Hecker, Maria Melchior, Ellenor Mittendorfer-Rutz, Marit Sijbrandij, Henrik Walter, Anke B. Witteveen, José Luis Ayuso-Mateos, María-Fe Bravo-Ortiz, Raffael Kalisch, Lara M.C. Puhlmann, Roberto Mediavilla

**Affiliations:** 1 Leibniz Institute for Resilience Research (LIR), Mainz, Germany; 2 Harvard TH Chan School of Public Health, CAUSALab, Boston, MA USA; 3 Department of Psychiatry, Icahn School of Medicine at Mount Sinai, New York, NY USA; 4 Department of Psychiatry, Clinical Psychology and Mental Health, La Paz University Hospital – IdiPAZ, Madrid, Spain; 5 Centro de Investigación Biomédica en Red de Salud Mental (CIBERSAM), Instituto de Salud Carlos III, Madrid, Spain; 6 Department of Environmental Health Sciences, Columbia University Mailman School of Public Health, New York City, NY USA; 7 Department of Personality, Assessment, and Psychological Treatment, University of Malaga - Biomedical Research Institute of Malaga (IBIMA), Málaga, Spain; 8 Faculty of Law, Education and Humanities, Universidad Europea de Madrid, Madrid, Spain; 9 Sorbonne Université, INSERM, Institut Pierre Louis d’Épidémiologie Et de Santé Publique, IPLESP, Equipe de Recherche en Epidémiologie Sociale, ERES, Paris, 75012 France; 10 Department of Clinical neuroscience, Division of Insurance medicine, Karolinska Institutet, Stockholm, Sweden; 11 Department of Clinical, Neuro- and Developmental Psychology, WHO Collaborating Center for Research and Dissemination of Psychological Interventions, Amsterdam Public Health Research Institute, Vrije Universiteit Amsterdam, Amsterdam, The Netherlands; 12 Department of Psychiatry and Neurosciences CCM, Charité - Universitätsmedizin Berlin, Corporate Member of Freie Universität Berlin, Humboldt-Universität Zu Berlin, Berlin Institute of Health, Berlin, Germany; 13 Department of Psychiatry, Universidad Autónoma de Madrid, Madrid, Spain; 14 Hospital Universitario La Princesa – IIS Princesa, Madrid, Spain; 15 Clinical Psychology and Behavioural Neuroscience, Faculty of Psychology, Technische Universität Dresden, Dresden, Germany; 16 Neuroimaging Center (NIC), Focus Program Translational Neuroscience (FTN), Johannes Gutenberg University Medical Center, Mainz, Germany

**Keywords:** Depression, Mental health, Stress, Occupational health, Trust, Social support, Health occupations, Psychology, Human behaviour

## Abstract

Healthcare workers (HCWs) in COVID-19 pandemic hotspots were exposed to heightened workplace stressor load. Structural occupational resilience factors could prevent work-related stressor exposure from translating into mental health problems but remain poorly understood. This study identifies resilience factors actionable at the workplace and examines the impact of prior stressor exposure early in the pandemic on the later development of depressive and general distress symptoms. We prospectively followed a convenience sample of HCWs working in Spain during the pandemic using a 3-wave online survey conducted in 2020 (wave 1, *n* = 2,422), 2021 (wave 2, *n* = 1,827), and 2022 (wave 3, *n* = 538). We operationalised resilience as low stressor reactivity (SR), quantified as individual deviations from the normative relation between stressors exposure and either depressive or distress symptoms. Mental health problems and stressor exposure both decreased over time, whereas stressor reactivity remained stable. Stressor exposure at baseline was inversely associated with resilience at follow-up. The structural occupational factors *support from colleagues*, *trust in the workplace*, and *perceived ability to recover from stress* were prospectively associated with resilience and thus identified as resilience factors. These results show that resilient responses of HCWs in times of crisis could be supported by promoting structural occupational resilience factors and mitigating cumulative stressor exposure. Future research should test this association in studies that allow causal inferences.

**Supplementary Information:**

The online version contains supplementary material available at 10.1038/s41598-025-09829-8.

## Introduction

The COVID-19 pandemic has had a profound and far-reaching impact across epicentres worldwide. In the European Union alone, 384,000 people had died from COVID-19 by the end of 2020—a number that had tripled by mid-2025^1^. For several weeks, healthcare workers (HCWs) in early hotspots were forced to adapt to this new reality without adequate training or protective equipment, and with limited access to usual coping mechanisms due to strict lockdowns and mobility restrictions. Unsurprisingly, HCWs in these regions have consistently reported elevated levels of mental health condition symptoms, particularly anxiety and depression, since the onset of the pandemic. Meta-analyses of non-representative cross-sectional studies have found that up to one third of HCWs reported clinically meaningful symptoms during the early phases of the pandemic^2–5^ with female HCWs and nurses being particularly affected. More recent longitudinal studies have found that many of these problems persisted for at least one year after the onset of the pandemic^6–9^. These issues were associated with an increased exposure to workplace-related stressors due to the pandemic, such as shortages of protective equipment, fear of infection, or involvement in triage decisions^10–14^.

The association between stressor exposure and concurrent mental health problems is in line with well-established findings in the general population, both before^15^ and during the COVID-19 pandemic^16–18^. Specifically, during the pandemic a large proportion showed worsened or moderate mental distress due to pandemic-related stressors^18^. Moreover, persistent or repeated stressor exposure can have a cumulative negative effect on future mental health, as suggested by prospective studies in both the general population^19^ and HCWs amidst the SARS and COVID-19 outbreaks^6,7,20,21^. These data align with the influential allostatic load model that describes the cumulative adaptation costs to stressor exposure^22^. In some cases, exposure to moderate amounts of adversity has conversely been found to decrease future negative effects of stressors, presumably via strengthening personal coping skills, in a process termed stress inoculation, resulting in a “growth” or “steeling effect”^23,24^. Exposure to stressors can serve as a challenge that causes individuals to develop novel skills, resources, and abilities to overcome it, which can subsequently also be applied to cope with future stressors^25^.

Even when exposed to severe or societal-level stressors such as the COVID-19 pandemic, it has been found that the most common response to adversity for about two-thirds of people in the general population is the maintenance or quick recovery of mental health^18,26–28^. However, this has recently been challenged, positioning that recovery is less common than previously believed^29,30^. This positive outcome is termed resilience^31^. Recent resilience models operationalize resilience as an outcome to adversity exposure, which results from a dynamic process of adaptation^31^.

Importantly, apparent individual differences in resilient outcomes can be confounded by underlying differences in the individual amount of stressor exposure^32^. To accurately estimate resilience outcomes, it is therefore necessary to account for the stressors experienced by individuals. Resilience can thus be operationalised as deviations from the normal relationship between the number of stressors people are exposed to and the mental health condition symptoms they report^32,33^. Within this framework, resilience outcomes are estimated using regression residuals of this normal relationship, termed stressor reactivity (SR) scores^32^. Lower SR scores then reflect relatively fewer mental health problems in response to the individually faced adversity than would be predicted on average, thus indicating resilience.

Even though several studies have previously identified resilience factors in HCWs^34,35^ hardly any of these investigations either accounted for individual differences in adversity exposure or focused on structural occupational resilience factors. The former is important for identifying resilience factors at least partly independently from exposure-related determinants of resilience and mental health –exposure to stressors and experiences of mental health problems can differ substantially even within the healthcare profession^36,37^. Most crucially, the latter helps identify both structural occupational and individual resilience factors to build resilient working environments and a resilient workforce^5^. For example, feeling supported by employers during the pandemic has been linked to a decreased state of anxiety and post-traumatic stress among HCWs^38^. At the same time, there is strong evidence that HCWs’ expectations and needs were not met during the pandemic^39^. The negative impact of these unmet needs, such as distress and isolation, as well as the interaction between organisational policies and interpersonal experience were identified by HCWs in qualitative research^37^. These data indicate the need for a better understanding of structural occupational resilience factors and thus inform policies.

The current study aims to address these research gaps to improve our understanding of HCWs’ resilience in times of crisis and identify structural occupational and person-level resilience factors. To that end, we used data from three assessment waves of a large-scale longitudinal dataset collected in Spain as part of an international prospective cohort study^13,40^. We quantified SR scores both in terms of general distress symptoms and depressive symptoms, since these were particularly prevalent among HCWs during the pandemic^41,^ and selected three potential resilience factors that are linked to the mental health of HCWs (*social support from colleagues* and *trust in the workplace*^37–39,42^) or improved stressor reactivity in the general population during the pandemic^16,43^ (*perceived ability to recover from stress*, REC). Our specific aims were to: (1) investigate changes in resilience (quantified as SR scores) over time; (2) test the potential stress inoculation or cumulative effect of baseline stressor exposure on current stressor reactivity; (3) test whether two potential structural occupational resilience factors, namely *support from colleagues* and *trust in the workplace*, and the individual resilience factor REC, are negatively associated with resilience; (4) analyse the moderating role of stressor exposure in the association between these resilience factors and resilience; and (5) estimate the mediating role of REC in the association between *support from colleagues* and resilience. The overall goal of this work was to inform decision-making and further resilience research on global health crises.

## Methods

### Study design and participants

The COVID-19 HEalth caRe wOrkErS (HEROES) project is a prospective cohort study that examines the mental health of HCWs following the initial pandemic outbreak in more than 20 countries^40^. Between March and June 2020, we developed an online survey that included mental health outcome measures and workplace- and COVID-19-related items designed ad hoc. The working group responsible for the survey included representatives from different countries, including Spain (RM, EF-J, and GM-A), and met every week over a 4-month period. Participants were HCWs recruited from outpatient and inpatient healthcare facilities, not necessarily involved in the care of COVID-19 patients, with or without clinical duties. In Spain, we used non-probabilistic sampling techniques to recruit volunteer participants. We collected self-reported data via an online survey in three different waves (wave 1 [baseline] from April 24th to June 22nd, 2020, *n* = 2,422 respondents; wave 2 from January 26th to March 8th, 2021, *n* = 1,827 respondents; and wave 3 from March 23rd to May 23rd, 2022, *n* = 538). These respectively concurred with the first pandemic outbreak, the third pandemic peak and the onset of vaccination campaigns among HCWs, and the fifth pandemic outbreak. The Ethics Committee at the Hospital Universitario La Paz approved the study before data collection started (identifier 4099) and all participants signed the informed consent form.

### Measures

#### Stressor exposure (E)

Stressor exposure (E) was calculated as the number of reported events that could be classified as workplace- and COVID-19-related stressors. Stressors were derived from a self-developed HEROES questionnaire, which included 193 items carefully designed to capture the unprecedented working situations of HCWs in Europe and Latin America (the full survey is available at https://osf.io/c73ts/). The HEROES study examined the relationship between these exposures and key mental health outcomes, such as symptoms of depression and suicidal thoughts, across various countries (e.g^11,13,14,44^). For this study, two raters (PP-R and RM) first chose all workplace- and COVID-19-related items that could be classified as workplace- and COVID-19-related stressors. This classification was based on similarity to items from established lists of daily stressors or hassles, namely the Mainz Inventory of Microstressors^45^ and the COVID-pandemic stressors lists used in the DynaCORE studies^43,46^ and on previously available evidence. The final stressor list comprised 17 items and is displayed in Supplementary Table 1 along with empirical supporting evidence. Items were recoded into dichotomous values (did occur/did not occur) and summarised as a total sum score of stressors. Since the questionnaire battery differed between waves, the sum of all stressors was divided by the sum of possible stressors, resulting in a proportional score, which is used as the measure of exposure to stressors (E).

#### Mental health problems (P)

Depressive symptoms were measured using the 9-item Patient Health Questionnaire (PHQ-9)^47^ specifically the Spanish version^48^. The PHQ-9 total score ranges from 0 to 27, with higher values indicating more depressive symptoms. The PHQ-9 displays satisfactory reliability (Cronbach’s alpha α = 0.86–0.89) and criterion and construct validity^47^. The Cronbach’s alpha for the full baseline sample was 0.88 (95% confidence interval from 0.87 to 0.89). General mental health problems were measured using the 12-item General Health Questionnaire (GHQ-12)^49^. The GHQ-12 ranges from 0 to 36, with higher scores denoting greater psychological distress. The scale has satisfactory reliability (Cronbach’s α = 0.79 to 0.91)^50^. In the full baseline sample Cronbach’s alpha α is 0.85 (95% CI 0.84, 0.86).

#### Stressor reactivity (SR) score

We calculated individual stressor reactivity (SR) as an estimate of outcome-based resilience, that is, the maintainance or quick recovery of mental health after exposure to stressors^32^. A mental health reaction to stressors is expected, however, the quantification of an individual’s reactivity in relation to the normative reaction to stressor exposure can indicate more or less resilient outcomes. To quantify resilience in the present study, the sample’s normative SR was computed by regressing mental health problems (P, assessed in two ways by the PHQ-9 or GHQ-12) against stressor exposure (E). First, a longitudinal normative E ~ P line was calculated using the subset of the sample based on complete cases (“complete case sample”, i.e., including only subjects that completed all 3 P assessments; PHQ-9 sample = 222; GHQ-12 sample = 234). We used this SR score to compare resilience patterns across waves.

For the main analyses, we used the entire available sample, including participants with < 3 assessments (i.e., all available data at each wave) to maximize data use. The cross-sectional relationship between E and P differed across waves (Pearson’s correlation coefficient E ~ PHQ-9: wave 1 [baseline]: r = 0.37, wave 2: r = 0.30, wave 3: r = 0.23; E ~ GHQ-12: wave 1 [baseline]: r = 0.33, wave 2: r = 0.24, wave 3: r = 0.14), presumably because samples differed substantially. We therefore calculated the normative E ~ P line separately for each cross-sectional wave in order to optimize E-P fits for the respective sample. Linear and quadratic model fits were compared using Chi-squared tests for each separate E ~ P line. The best fitting regression models were used for the deriving of the E-P line across waves in the complete-cases sample (see Supplementary Fig. 1) and separately at each wave in the full sample (see Supplementary Fig. 2). These E-P lines show participants’ normatively expected P given their level of E. Individual SR scores were then quantified as the difference between the expected P and their observed P (i.e., the residual to the E-P line), resulting in one PHQ-9-based SR score (SR_dep_) and a GHQ-12-based SR score (SR_dis_), in line with previous approaches^42^. Within this framework, a positive SR score denotes an “over”-reaction to stressors, and a negative SR score an “under”-reaction, always compared to the sample’s normative stressor reactivity. SR scores are therefore an estimate of resilience, where negative scores indicate a better ability to maintain mental health after stressor exposure.

### Resilience factors

From the extensive survey data, we identified two potential structural occupational resilience factors that could be acted on at the organisational level. These factors were measured by single items that were created specifically for the purpose of the study (see^40^). *Social support from colleagues* in the workplace was assessed with a single item (“I have a reliable network of supportive colleagues at work”) to which participants indicated their agreement on a 4-point Likert scale (“strongly disagree [1]” to “strongly agree [4]”). *Trust in the workplace* was measured with the item “To what extent do you trust that your workplace (its leadership) can manage the COVID-19 pandemic?”. Answer options were on a 5-point Likert scale from “not at all (0)” to “extremely (4)”.

In addition to structural occupational resilience factors, we used the 6-item Brief Resilience Scale^51^ specifically the Spanish version^52^ to measure the perceived ability to recover from stress (REC). The REC items ask the participants to report their general ability to recover from stress and offer rating scales from 1 (“strongly disagree” for direct items and “strongly agree” for inverse items) to 5 (“strongly agree” for direct items and “strongly disagree” for inverse items). Ratings are summed and final scores range from 0 to 6. The Cronbach’s alpha for the full sample at baseline was 0.80 (95% confidence interval from 0.78 to 0.81).

### Data availability

**Fig. 1:**
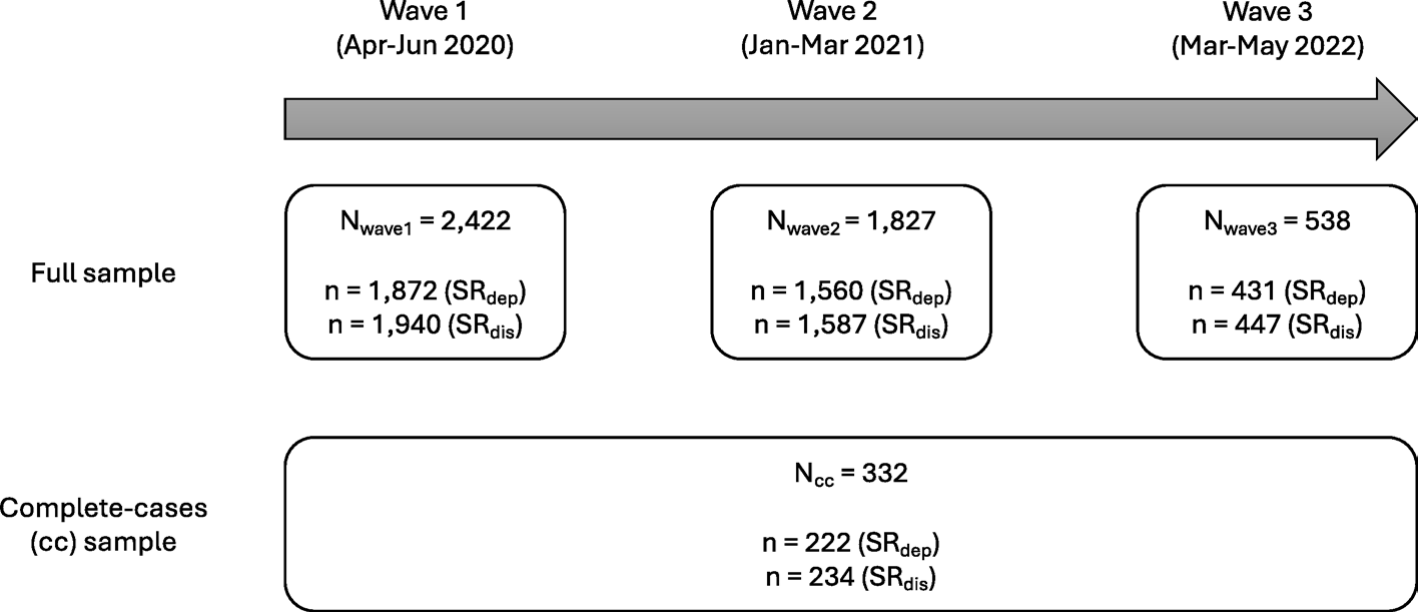
Number of respondents across waves. The survey was open to new respondents for each wave and the respondents could drop out at any point. This explains the differences in the observations across waves and outcomes. To calculate stressor reactivity (SR) scores, both a stressor exposure (E) score, and a mental health score, as measured by the Patient Health Questionnaire PHQ-9 (SR_dep_) or the General Health Questionnaire GHQ-12 (SR_dis_), were required.

Using the available data, we computed SR scores that served as estimates of resilience (see above). Final cross-sectional samples with SR_dep_ consisted of *n* = 1,872, *n* = 1,560, and *n* = 431 at waves 1, 2, and 3, respectively. Cross-sectional samples with SR_dis_ consisted of *n* = 1,940, *n* = 1,587, and *n* = 447 respondents at waves 1, 2, and 3, respectively. For longitudinal samples, stress and mental health data were available for *n* = 222 (SR_dep_) and *n* = 234 (SR_dis_) participants in all waves. Data availability is summarised in Fig. 1. Detailed descriptions of the full sample at baseline and the first follow-up assessment have been published elsewhere (for wave 1^13^ and for wave 2^56^).

#### Statistical analyses

We first ran univariate linear regression models on the complete-cases sample to explore whether stressor exposure, mental health problems, and finally SR, changed across waves (i.e., resilience over time). The complete-cases sample was used in order to be sensitive to changes over time. For the main analyses, we used the full sample, in order to maximize data use. We computed regression models to examine the effect of stressor exposure at baseline (wave 1) on cross-sectional SR scores at follow-up (waves 2 and 3) in the full sample. We tested both linear and curvilinear associations by adding a quadratic term.

Furthermore, we tested the association between (a) *support from colleagues* and *trust in the workplace*, and REC measured at baseline (resilience factors) and (b) the SR score at follow-up, again on the full sample. Both linear regression models included age and gender as covariates. The relationship between resilience factors was examined in correlations, as well as a multi-variate analysis predicting SR scores including all resilience factors and baseline E exposure. Additionally, moderating effects of stressor exposure on the association between each resilience factor and stressor reactivity were tested in linear regression models.

Lastly, cross-sectional mediation analyses following Baron and Kenny^54^ were conducted with *support from colleagues* as the exposure, REC as the mediator, and the SR scores as the outcome. In short, the Baron and Kenny method seeks to quantify the two paths between exposure and outcome (one, independently of the mediator, and another one through the mediator) via three regressions: (i) exposure predicting the outcome, (ii) exposure predicting the mediator, and (iii) exposure and mediator predicting the outcome. A common interpretation is that partial mediation exists if the strength of the association between exposure and outcome is reduced after controlling for the mediator. We chose this widely adopted approach given that there was no evidence of an interaction between *support from colleagues* and REC, and under the assumption of no reverse causation between REC and *support* *from colleagues*. Bootstrapped indirect effects are reported (5000 iterations). Sensitivity analyses were conducted on the full longitudinal sample.

All analyses were performed analogously with the outcomes SR_dep_ and SR_dis_, and were carried out using R 4.3.0 and R Studio. All estimates were reported as regression coefficients along with 95% CIs. The analytic code, dataset, and codebook are available elsewhere (https://osf.io/c73ts/).

## Results

At baseline, the cross-sectional sample (*N* = 2,422) consisted mostly of female HCWs (*n* = 1,871, 78%) mainly working as physicians (*n* = 757, 34%) or nurses (*n* = 730, 32%). These characteristics were similar in the complete-cases sample (*n* = 332), where 82% were female, 36% were physicians, and one third were nurses. Using cutoff values described in the literature^55^ (PHQ-9 total score equal or higher than 10 points), nearly one-third of the sample reported symptoms compatible with depressive disorders (full sample: *n* = 507, 27%, complete-cases sample: *n* = 89, 30%). Their baseline sociodemographic characteristics, workplace- and COVID-19-related exposures, and mental health outcome scores are shown in Table 1^58^.

**Table 1:** Baseline characteristics of the participants included in the full sample (N_wave1_ = 2,422) and in the complete-case sample (N_cc_ = 332).

Characteristic	Full sample, *N* = 2,422^1^	Complete-cases sample, *N* = 332^1^	Lost-to-follow-up, *N* = 2,090^1^	*p*-value^2^
Age, M (SD)	43 (12)	43 (11)	43 (13)	0.5
Unknown, n	96	4	92	
Gender, n (%)				0.14
Male	514 (22%)	61 (18%)	453 (22%)	
Female	1,871 (78%)	270 (82%)	1,601 (78%)	
Unknown	37	1	36	
Profession				0.5
Ancillary worker	138 (6.1%)	18 (5.8%)	120 (6.2%)	
Health technician	206 (9.1%)	21 (6.7%)	185 (9.5%)	
Nurse	730 (32%)	104 (33%)	626 (32%)	
Other	194 (8.6%)	22 (7.0%)	172 (8.9%)	
Other HCW	229 (10%)	35 (11%)	194 (10.0%)	
Physician	757 (34%)	113 (36%)	644 (33%)	
Unknown	168	19	149	
E, M (SD)	39 (15)	39 (15)	39 (15)	0.5
Unknown, n	157	16	141	
Support from colleagues, M (SD)	3.23 (0.74)	3.26 (0.68)	3.23 (0.75)	0.7
Unknown, n	549	37	512	
Trust in the workplace, M (SD)	2.26 (0.99)	2.35 (1.03)	2.25 (0.98)	0.040
Unknown, n	282	21	261	
REC, M (SD)	3.19 (0.72)	3.17 (0.73)	3.20 (0.72)	0.6
Unknown, n	574	39	535	
PHQ-9 dichotomous, n (%)				0.2
No MDD	507 (27%)	89 (30%)	418 (27%)	
Probable MDD	1,365 (73%)	207 (70%)	1,158 (73%)	
Unknown	550	36	514	
PHQ-9 total, M (SD)	7.3 (5.4)	7.6 (5.4)	7.3 (5.4)	0.3
Unknown, n	550	36	514	
GHQ-12 total, M (SD)	15.8 (6.1)	16.1 (6.0)	15.7 (6.1)	0.2
Unknown, n	482	30	452	

*M = Mean*,* SD = Standard Deviation*,* REC = perceived ability to recover from stress*,* PHQ-9 = 9-item Patient Health Questionnaire*,* MDD = major depressive disorder*,* GHQ-12 = General Health Questionnaire*.

### Resilience across the waves of the pandemic

**Fig. 2:**
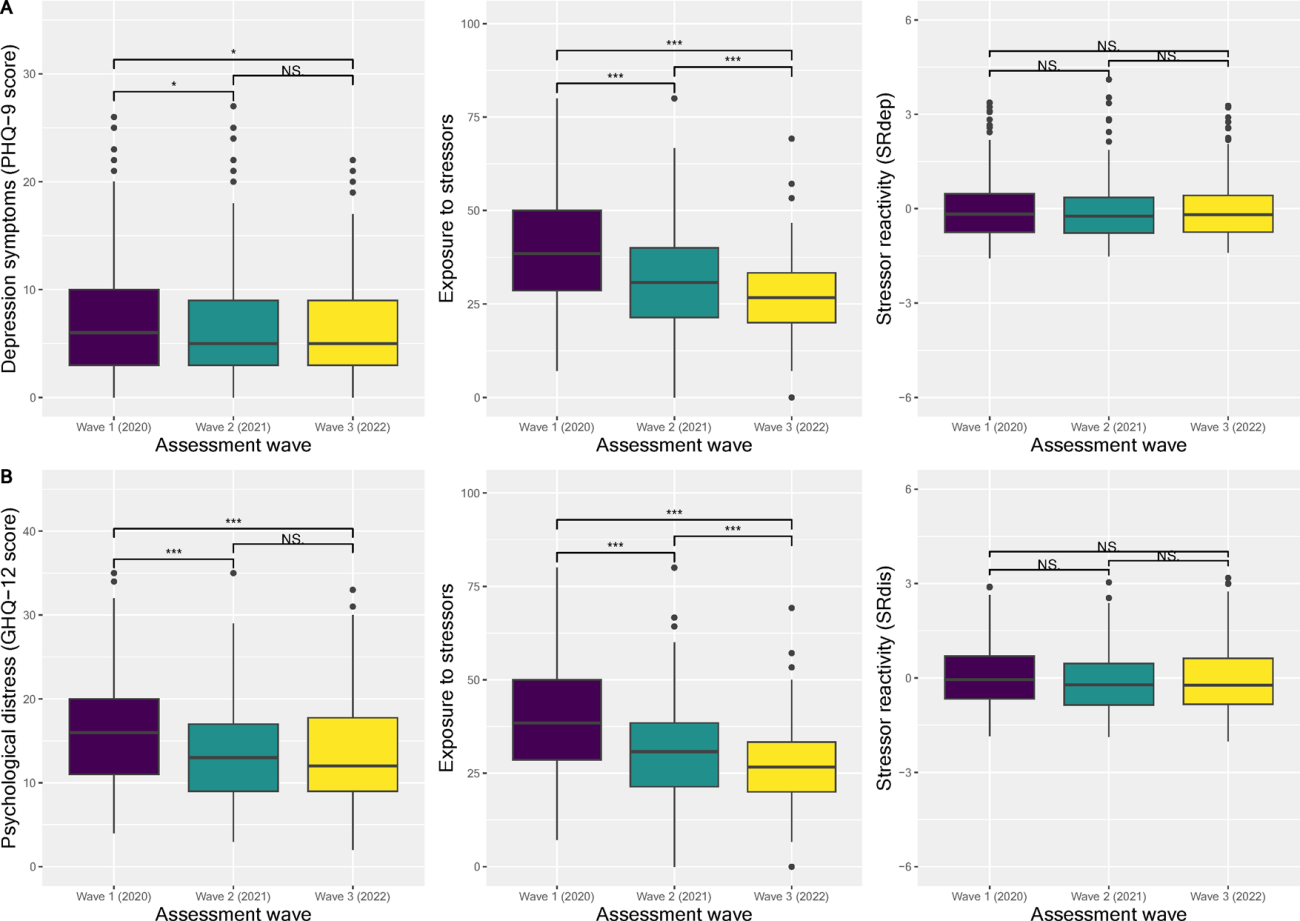
Changes in mental health problems (P), depression symptoms (PHQ-9), and psychological distress (GHQ-12 or PHQ-9). Exposure to stressors (E), and stressor reactivity (SR) over time in the complete-cases sample (N_complete-cases_ = 332). SR scores were computed using the 9-item Patient Health Questionnaire (PHQ-9) for depressive symptoms (SR_dep_, panel A) and the 12-item General Health Questionnaire (GHQ-12) for psychological distress (SR_dis_, panel B). To compute SR scores, E and P had to be available for each individual at all waves (panel A: *n* = 222, panel B: *n* = 234), hence the slight differences in E scores between panels.

Respondents in the complete-cases sample reported on average fewer symptoms of depression and psychological distress and less exposure to stressors over time. Notably, however, average SR_dep_ and SR_dis_ scores (calculated across all measurement waves to allow longitudinal comparison) remained stable (see Fig. 2), and showed a moderate-to-high correlation at each wave (r_wave 1_= 0.68, r_wave 2_= 0.76, r_wave 3_= 0.70). The time effect estimates, and their 95% confidence intervals are shown in Supplementary Table 2.

### Effect of stressor exposure during the initial pandemic outbreak

**Fig. 3:**
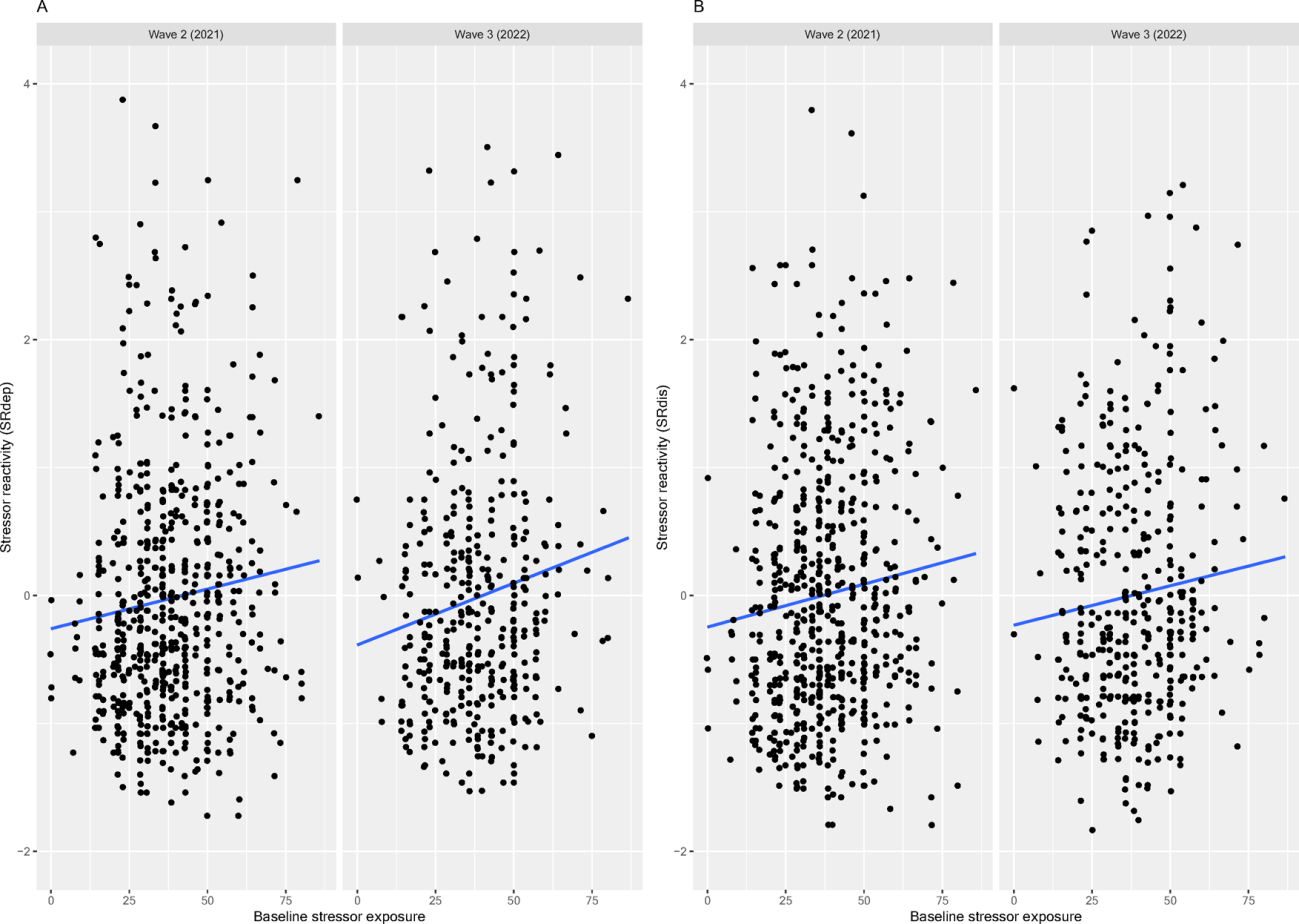
Association between stressor exposure (E) at baseline and stressor reactivity (SR) scores at follow-up in the full sample. Depression SR scores (SR_dep_, panel A) are computed using the 9-item Patient Health Questionnaire (PHQ-9) and general distress SR scores (SR_dis_, panel B) are computed using the 12-item General Health Questionnaire (GHQ-12).

We tested the association between stressor exposure (E) at baseline (April-June 2020) and cross-sectional SR at follow-up waves in the full sample. The distribution of E at baseline was normal and included low scores (i.e., values down to 0 stressors) despite sampling from a highly stressor exposed population (see Supplementary Fig. 3), indicating that there was individual variance in stressor exposure. The effect of E at wave 1 (baseline) on SR_dep_ at waves 2 (2021) and 3 (2022) was significant (B = 0.10 [*p* = 0.0247] and B = 0.015 [*p* = 0.00971], respectively) (see Fig. 3). The effect of baseline E and SR_dis_ was significant at wave 2 (B = 0.10, *p* = 0.0287), but only marginal at wave 3 (B = 0.09, *p* = 0.0960). As per design, there was no interaction effect of E at baseline and wave 1, since the SR score was calculated cross-sectionally and SR at wave 1 fully accounted for E at wave 1. In the reduced sample based on complete longitudinal cases, the effect of baseline stressor exposure on SR went in the same direction, but did not reach significance (see Supplementary Fig. 4).

### Resilience factors

We tested whether the potential structural occupational resilience factors assessed at study baseline (wave 1) were prospectively associated with cross-sectional SR scores at the follow-ups. We found that reporting *support from colleagues* and *trust in the workplace* was associated with both lower SR_dep_ and SR_dis_, especially at wave 2. Looking at the personal resilience factor, we found that REC was inversely associated with SR at wave 2 (see Table 2). In the complete cases sample, all the trends were replicated but not all effects were statistically significant (see Supplementary Table 3).

**Table 2:** Concurrent (wave 1) and prospective (waves 2 and 3) associations between resilience factors at baseline and stressor reactivity (SR) scores at baseline and follow-up in the full sample.

	Wave 1 (2020)		Wave 2 (2021)		Wave 3 (2022)	
	Unadjusted B (95% CI)	Adjusted B (95% CI)	Unadjusted B (95% CI)	Adjusted B (95% CI)	Unadjusted B (95% CI)	Adjusted B (95% CI)
SR_dep_						
Support from colleagues	−0.22 (−0.28, −0.16)	−0.22 (−0.28, −0.16)	−0.23 (−0.34, −0.12)	−0.22 (−0.33, −0.11)	−0.1 (−0.24, 0.03)	−0.12 (−0.26, 0.02)
Trust in the workplace	−0.11 (−0.16, −0.07)	−0.1 (−0.15, −0.06)	−0.11 (−0.19, −0.03)	−0.09 (−0.17, −0.01)	−0.12 (−0.21, −0.02)	−0.14 (−0.24, −0.04)
REC	−0.54 (−0.6, −0.48)	−0.52 (−0.58, −0.46)	−0.45 (−0.55, −0.34)	−0.43 (−0.54, −0.32)	−0.38 (−0.52, −0.25)	−0.39 (−0.52, −0.25)
SR_dis_						
Support from colleagues	−0.23 (−0.29, −0.17)	−0.23 (−0.29, −0.16)	−0.19 (−0.3, −0.08)	−0.19 (−0.29, −0.08)	−0.17 (−0.3, −0.04)	−0.16 (−0.29, −0.02)
Trust in the workplace	−0.13 (−0.17, −0.09)	−0.12 (−0.17, −0.07)	−0.09 (−0.17, −0.02)	−0.09 (−0.16, −0.01)	−0.16 (−0.26, −0.07)	−0.18 (−0.27, −0.08)
REC	−0.54 (−0.6, −0.48)	−0.53 (−0.59, −0.47)	−0.35 (−0.45, −0.24)	−0.35 (−0.45, −0.24)	−0.29 (−0.43, −0.16)	−0.3 (−0.44, −0.16)

Estimates of beta-coefficients in the adjusted models were corrected for age and gender. REC = perceived ability to recover from stress. SRdep = SR score computed using depressive symptoms as measured by the 9-item Patient Health Questionnaire (PHQ-9). SRdis = SR score computed using psychological distress symptoms as measured by the 12-item General Health Questionnaire (GHQ-12).

Overall, the personal resilience factor REC showed greater associations with SR in waves 2 and 3 than structural occupational resilience factors and was only moderately correlated with these factors (Supplementary Table 4). In a multivariate regression including all resilience factors and stressor exposure at baseline, it emerged that the structural occupational resilience factors still explained unique variance at different waves over and beyond REC. For both SR_dep_ and SR_dis_, *support from colleagues* was a significant predictor in wave 2 but not in wave 3, whereas *trust in the workplace* was significant in wave 3 for ﻿SR_dis_ only. In contrast, baseline stressor exposure did not explained additional unique variance at any wave (see Table 3).

**Table 3:** The multivariate linear regression model in the full sample, including resilience factors *trust in the workplace*, *support from colleagues*, and REC, as well as stressor exposure, all measured at baseline.

	Dependent variable			
	SR_dep_		SR_dis_	
	Wave 2	Wave 3	Wave 2	Wave 3
Support from colleagues	**−0.163** ^**^	0.074	**−0.113** ^*^	−0.018
	(0.057)	(0.064)	(0.057)	(0.067)
Trust	−0.016	**−0.087**	−0.013	**−0.151** ^**^
	(0.040)	(0.048)	(0.041)	(0.050)
REC	**−0.424** ^***^	**−0.531** ^***^	**−0.421** ^***^	**−0.398** ^***^
	(0.055)	(0.062)	(0.055)	(0.064)
Stressor exposure	0.002	0.005	0.002	0.001
	(0.003)	(0.003)	(0.003)	(0.003)
Constant	**1.817** ^***^	**1.364** ^***^	**1.655** ^***^	**1.588** ^***^
	(0.268)	(0.305)	(0.269)	(0.319)
Observations	567	353	558	352
R^2^	0.137	0.206	0.123	0.141
Adjusted R^2^	0.131	0.197	0.117	0.131
Residual Std. Error	0.917 (df = 562)	0.875 (df = 348)	0.918 (df = 553)	0.914 (df = 347)
F Statistic	**22.282** ^***^ (df = 4; 562)	**22.563** ^***^ (df = 4; 348)	**19.434** ^***^ (df = 4; 553)	**14.285** ^***^ (df = 4; 347)

*Perceived ability to recover from stress (REC)*,* df = degrees of freedom* * *p < 0.05*, ** *p < 0.01*, *** *p < 0.001*

### Interactions between baseline exposure and resilience factors

**Fig. 4:**
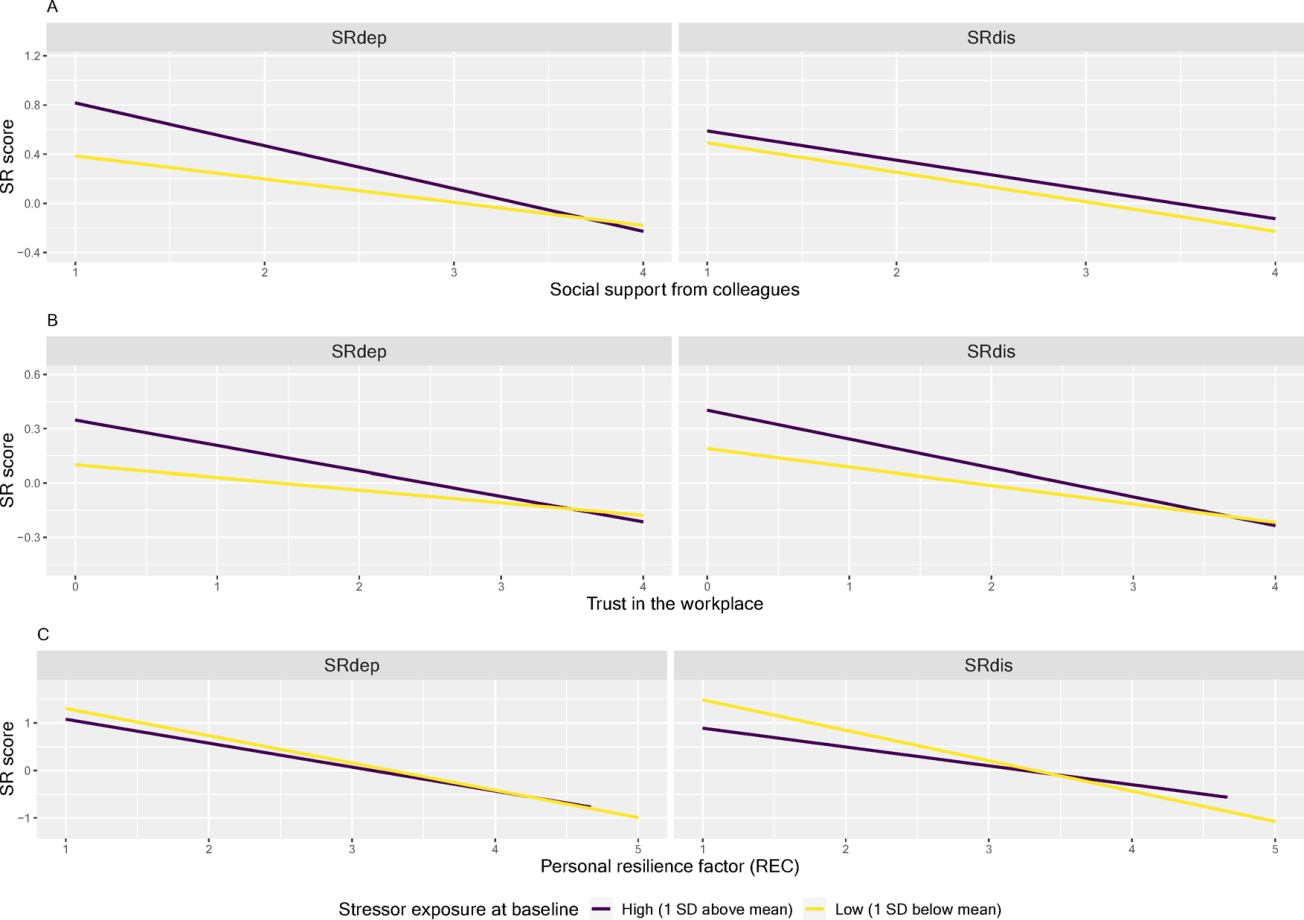
Association between the resilience factors in the full sample. *Social support from colleagues* (panel A), *trust in the workplace* (panel B), and the *perceived ability to recover from stress* (REC, panel C), and the stressor reactivity scores (SRs) computed using self-reported depressive symptoms (SR_dep_, left) and symptoms of psychological distress (SR_dis_, right). To show the moderation effects of baseline stressor exposure (E), we present the linear regression estimates and standard errors for respondents with E scores that are one standard deviation (SD) above (purple line) or below (yellow line) the sample mean. Interactions between resilience factors and baseline E are significant (*p* < 0.05) in panel B (SR_dep_: B = − 0.003, *p* = 0.03; SR_dis_: 0.003, *p* = 0.03) and panel C, right (SR_dis_: B = 0.004, *p* = 0.02). The full model results are in Supplementary Table 5.

Examining interactions between individual stressor exposure and resilience factors, the effect of *support from colleagues* on SR_dep_ or SR_dis_ was independent of baseline E (SR_dep_: B = −0.0031, *p* = 0.0812; SR_dis_: B = −0.00041, *p* = 0.8199). In contrast, the effect of *trust in the workplace* on both SR scores was moderated by baseline E (SR_dep_ B = −0.0027, *p* = 0.03163; SR_dis_: B = −0.0027, *p* = 0.0287), indicating that the higher E at baseline, the greater the effect of levels of *trust in the workplace* on SR (see Fig. 2). Baseline E also moderated the effect of REC on SR_dis_, but not on SR_dep_ (see Fig. 4).

### The mediating role of REC

Exploring potential effect pathways, REC mediated the effect of *support from colleagues* on SR_dep_ (Mean bootstrapped indirect effect = −0.16 (95% CI −0.27,−0.07, *p* < 0.0001), as well as on SR_dis_ (Mean bootstrapped indirect effect = −0.13 (95% CI −0.22,−0.06, *p* < 0.0001).

## Discussion

We followed a sample of healthcare workers (HCWs) from an early pandemic hotspot over a 2-year period after the initial COVID-19 pandemic outbreak. We measured stressor exposure (E) and depressive symptoms (P) at each wave, and used the residuals of the E-P regression line to estimate stressor reactivity (SR) scores as an estimate of resilience^32^. In contrast to mental health condition symptoms, SR scores on average remained stable over time. Baseline stressor exposure was inversely associated with resilience at follow-ups, which suggests a probable cumulative effect of stressor exposure. Furthermore, we identified three resilience factors prospectively associated with resilience, namely, *social support from colleagues*, *trust in the workplace*, and *perceived ability to recover from stress* (REC). *Trust in the workplace* was particularly important for individuals who experienced high levels of baseline stressor exposure. Moreover, people with low self-assessed stress recovery were more reactive even to low levels of stressors. Additionally, the effect of *support from colleagues* on stressor reactivity was mediated by higher REC.

### Resilience across waves of the pandemic

A strong association between adversity and general mental health problems and depressive symptoms is well-established^15,32,56^ and was replicated with our measure of individual stressor exposure here. We found that, while mental health condition symptoms (both depressive and distress symptoms) and stressor exposure decreased on average over the first two years of the pandemic, SR as an estimate of outcome-based resilience remained stable. This suggests that apparent decreases in mental health problems over the course of the pandemic were in fact fully accounted for by changes in stressor exposure.

By adjusting for apparent systematic changes in individual stressor exposure across pandemic waves, we avoided confounded estimates of resilience factors^32^. We are not aware of any previous longitudinal studies among HCWs that approached resilience by accounting for individual E. However, this approach has been successfully used in a cross-sectional sample of psychotherapists to identify resilience factors in the first phase of the pandemic. Zerban and colleagues^42^ identified resilience factors associated with both profession-specific and general SR, including perceived social support, REC, optimism, self-compassion, self-efficacy, mentalising, and compassion satisfaction.

Researchers may consider similar adjustments for individual stressor exposure in their models to distinguish resilience from neighbouring concepts such as mental health or wellbeing, especially when studies involve diverse populations of HCWs exposed to different levels and types of stressors^20,42,57^. This also avoids misinterpreting systematic changes in P as changes in resilience, when they are in fact trivially explained by changes in E.

### Effect of stressor exposure during the initial pandemic outbreak

It is an organisational responsibility to avoid the presumably particularly deleterious effects of continued or repeated work-stressor overload. Here, we show that prior stressor exposure negatively affects HCWs’ subsequent resilience. That is, HCWs exposed to a higher number of stressors in the early weeks of the pandemic (study baseline/wave 1) reacted more strongly with depressive symptoms to stressors (i.e., showed less resilient outcomes) in waves 2 and 3 (one and two years later, respectively).

In regard to resilience to general distress, the picture was less clear, as the negative effect of baseline stressor exposure on resilience to general distress symptoms could only be seen in wave 2 and not in wave 3. This could indicate a more long-term effect of stressor exposure on depressive symptoms than general distress. Similarly, a cross-sectional analysis of this sample at wave 1 found stronger effects of single stressors on depressive symptoms than psychological distress^13^. These data suggest that experiencing stressors during the pandemic led to a specific increase in depressive symptoms more than in general distress, which also aligns with observed increases specifically in anxiety and depressive disorders as a result of the pandemic^4,5^. However, this could also be explained by a stronger impact of stressors on depressive symptoms. Overall, the effects of baseline E were small but relatively consistent. These results support the concept of cumulative effect of stressor load, in which prior or continued exposure to adversity is associated with greater reactivity to future stressors^19,58,59^. We found no evidence for non-linear relationships between baseline E and resilience, which could have suggested the existence of an optimal amount of stressor exposure that supports coping with subsequent stressors in a sort of “steeling effect”, in line with the stress inoculation theory^24,25,60^. Whereas the cumulative effect tapered off, this may be a function of the reduced sample size in wave 3.

Research shows that prior stressor exposure can increase the risk of experiencing mental health problems^19,58^. This is especially the case for uncontrollable stress (as exemplified by human and animal lab studies, e.g^61^^[,62^;) and stressors that are appraised as hindrances or threats, rather than challenges^63,64^. Additionally, chronic stress exposure is robustly linked to particularly deleterious health outcomes^65^. HCWs in qualitative interviews conducted in Spain and the US during the pandemic described very difficult, challenging, and novel experiences, and in the case of the US sample, reported that the effect of long-term exposure further exacerbated negative outcomes^37,66^. Chronicity of stressor exposure may therefore explain an absence of inoculating stress effects.

### Resilience factors

We identified two structural occupational predictors of resilience that are actionable at an organisational level, *support from colleagues* and *trust in the workplace*, and one personal resilience factor, REC. The structural occupational resilience factors were prospectively and directly associated with resilience at least one year after the initial pandemic outbreak. Because *trust in the workplace* was measured during the initial pandemic outbreak, it is likely that it reflects whether HCWs perceived their institution as prepared to support HCWs in the frontline, not only by providing protective equipment such as gloves and masks, but also by ensuring adequate working conditions (i.e., sufficient staff that allowed HCWs to rest properly after long and exhausting shifts). Our findings are therefore in line with COVID-19 studies linking good mental health with provision of protective equipment^11^ and adequate working conditions among HCWs^44,67^ and with reports of positive associations between institutional trust and mental health in the general population^68^ and among HCWs^44^.

On the other hand, the finding that social support is associated with good mental health outcomes is well-established^69–71^ and has been replicated in many samples of HCWs during the COVID-19 pandemic^17,42,72,73^. Perceived social support has also been consistently associated with lower stressor reactivity^18,42^. Here, we provide additional estimates of prospective associations of perceived social support specifically at the workplace and resilience (as estimated by stressor reactivity) in HCWs. This adjustment provides less biased estimates and supports strategies to promote social networks at the workplace.

Lastly, our results are in line with previous studies that show that perceived ability to recover from stress is linked with decreased reactivity to stressors^16,43^. Our results offer additional insights by showing that this self-perception continues to be important in high stress exposure healthcare environments and appears to partially capture different aspects of resilient response than structural occupational factors.

### Interactions between baseline exposure and resilience factors

Compared to the commonly strong correlations between personal resilience factors (e.g., Veer and colleagues^43^ r_s_ 0.03-0.54, in absolute values), the resilience factors assessed in this study were only weakly correlated, especially the personal resilience factor with institutional resilience factors (r_s_ 0.12, 0.14). In a combined multivariate regression, both types of resilience factors explained unique variance, improving the overall explained variance of the model. This suggests that structural occupational resilience factors may capture important variance over and above personal resilience factors, even when only assessed via self-report. We suggest that future studies on HCW wellbeing measure them in addition to person-level resilience factors.

*Trust in the workplace* had a stronger protective association at higher levels of baseline stressor exposure. This indicates that *trust in the workplace* is particularly important at high levels of individual burden, for example because it can offset the negative effects of past stress exposure as a risk factor to some extent. Relatedly, baseline stressor exposure was negatively correlated with trust, indicating that greater exposure at the beginning of the pandemic may have happened at institutions that were also perceived as less trustworthy, or alternatively, that exposure eroded trust in the institution.

The same interaction pattern was not found for *support from colleagues*, showing that perceived social support is similarly important at each level of baseline stressor exposure. Self-perceived stress recovery on the other hand appeared to be most important at lower levels of stressor exposure. Individuals with low REC paired with low baseline stressor exposure still had higher stressor reactivity across all waves than those with high levels of baseline stressor exposure. A possible explanation for this result is that those who perceive their ability to recover as low are particularly at risk of having high reactivity even at low level of exposure. However, this pattern was only found with resilience to general distress symptoms and not to depressive symptoms.

Overall, given that the effects of baseline exposure became non-significant in the joined model with all resilience factors, it is possible that baseline exposure mostly influenced stressor reactivity by means of negatively influencing self-perceived resilience factors.

Apart from this result, we observed almost identical effects of resilience factors on both SR measures, based on depression or general distress scores. A small difference in effects of baseline E suggests that past exposure may be more likely to increase subsequent stressor reactivity with depressive symptoms. This suggests that these factors are general, in that they are associated with resilience to a wider range of mental health problems (see also^42^).

### The mediating role of REC

We also found that the effect of *support from colleagues* on stressor reactivity was mediated by REC. This is in line with previous research that positions perceived good stress recovery closer to a resilient outcome, influenced by other resilience factors^31^. Prior evidence shows that REC mediates the effect the resilience factor “positive appraisal style of stressors” on stressor reactivity^16,42,43^ and that the effect of *support from colleagues* is mediated in turn by positive appraisal style. Presumably, talking with other persons one is close to can help an individual see stressful situations more positively, leading them to also perceive themselves as more resilient, and consequently, lowering stressor reactivity. Our results indicate that the mediation can also be shown directly between *support* from colleagues and REC without the intermediate mediator of positive appraisal style. In other words, (perceived) social support may help individuals engage in positive appraisals of stressors and see themselves as better able to recover from stressors.

### Implications

The World Health Organization and the International Labour Organization call for stakeholders and employers to prevent exposure to psychosocial risks at work, protect and promote mental health and wellbeing, and support people with mental health conditions^74^. Our findings have implications for each stage. We found that one in five HCWs have clinically elevated levels of depressive symptoms two years after the initial pandemic outbreak, emphasising the need to support people with mental health conditions at work. We showed that exposure to work-related stressors during the initial pandemic outbreak has a negative impact on subsequent resilience one and two years later. This pattern indicates the cumulative risk of long-term stressor exposure at the workplace. It is therefore imperative that workplaces reduce stressor load with early structural and preventive interventions.

Furthermore, we identified two structurally actionable areas that might help promote mental health and wellbeing at work: trustworthiness and peer support. We thus call for workplaces to promote trust among their workers by ensuring transparent and frequent communications, even when information or evidence are unclear or even contradictory. This is particularly important in highly stressor exposed groups, as institutional trust becomes even more important at high levels of past exposure and because the detrimental effects of exposure may be associated with eroding institutional trust. In addition to this, healthcare settings must provide spaces for peer support, either digital or face-to-face. Our results further highlight the importance of self-perceived stress recovery as both a moderator of the effect of stressors as well as a mediator of the effect of *support from colleagues*. It could therefore serve as a good monitor for identifying vulnerable groups. Considering the different levels of distress and the increasing needs as people become more distressed, we call for offering evidence-based scalable interventions in a stepped-care format^75,76^.

### Strengths and limitations

This study benefitted from large samples of HCWs highly exposed to stressors at each wave, an important and vulnerable study population, as well as an unusually long follow-up period. Furthermore, it used a robust and tested method to estimate outcome-based resilience. The inclusion of both organisational and personal resilience factors enhanced the scope of our conclusions and allowed for the identification of actions at both the individual and institutional level.

Nevertheless, this study has some limitations. First, we used non-probabilistic sampling techniques that increased the risk of selection bias in our sample. Although our participants are similar to those included in larger and more representative studies conducted in Spain^10^ the generalisation of our results may be hampered. Second, many participants were lost to follow-up, which may have also led to selection bias. Third, these initial results do not allow for sound causal inferences. Studies focusing on well-defined interventions and RCT to improve resilience via the pathways identified here may provide stronger evidence to inform policymaking. Fourth, structural occupational resilience factors were assessed only at the individual level, rather than at the institutional level (such as, retention, supervision, staff rooms), rendering them vulnerable to subjective biases. Lastly, the stressors in this study have been defined and recoded *post hoc* and were not designed to be part of the operationalisation of outcome-based resilience. The stressor list is thus probably not exhaustive of all work-related stressors faced by HCWs. Nevertheless, the classification and recoding of stressors was based on several studies that used similar stressor lists^42,43,45,46^. Additionally, the considerable association between most of these stressors and mental health outcomes have been systematically reported across countries within the research consortium^11,13,14,44,53,77–80^.

### Conclusion and future directions

Overall, our work contributes to a better understanding of structural occupational resilience mechanisms for HCWs in times of crisis and in particular the long-term consequences of the COVID-19 pandemic. Leveraging a large dataset and new resilience research methods, we call for stakeholders to activate social support and trust and to minimise exposure to stressors early in order to promote resilience in working environments with high stressor exposure. Future research should expand on these results by systematically identifying the most important stressors for HCWs, developing measures of resilience factors at the institutional level, and studying both in representative observational studies that are ideally integrated in daily clinical routine, thus limiting dropouts.

## Electronic supplementary material

Below is the link to the electronic supplementary material.

Supplementary Material 1

## Data Availability

A reduced and anonymised dataset can be accessed in Open Science Framework along with the data dictionaries and the analytic code (https://osf.io/c73ts/).
