# Supplementary material for: Resilience changes and occupational resilience factors among healthcare workers during and after the COVID-19 pandemic: A 2-year prospective cohort study
Source: Sci Rep. 2025 Aug 10;15:29263. doi: 10.1038/s41598-025-09829-8 (PMC12336339; doi:10.1038/s41598-025-09829-8)
Supplement: Supplementary file 1 — Supplementary Material 1 [file 41598_2025_9829_MOESM1_ESM.pdf]

Resilience changes and occupational resilience factors among healthcare workers during and after the COVID-19 pandemic: a 2-year prospective cohort study

Supplementary Figures and Tables

Supplementary Figure 1 .... ***2***

Supplementary Figure 2 .... ***3***

Supplementary Figure 3 .... ***4***

Supplementary Figure 4 .... ***5***

Supplementary Table 1 .... ***6***

Supplementary Table 2 .... ***8***

Supplementary Table 3 .... *Error! Bookmark not defined.*

Supplementary Table 4 .... ***9***

Supplementary Table 5 .... ***11***

Supplementary Figure 1

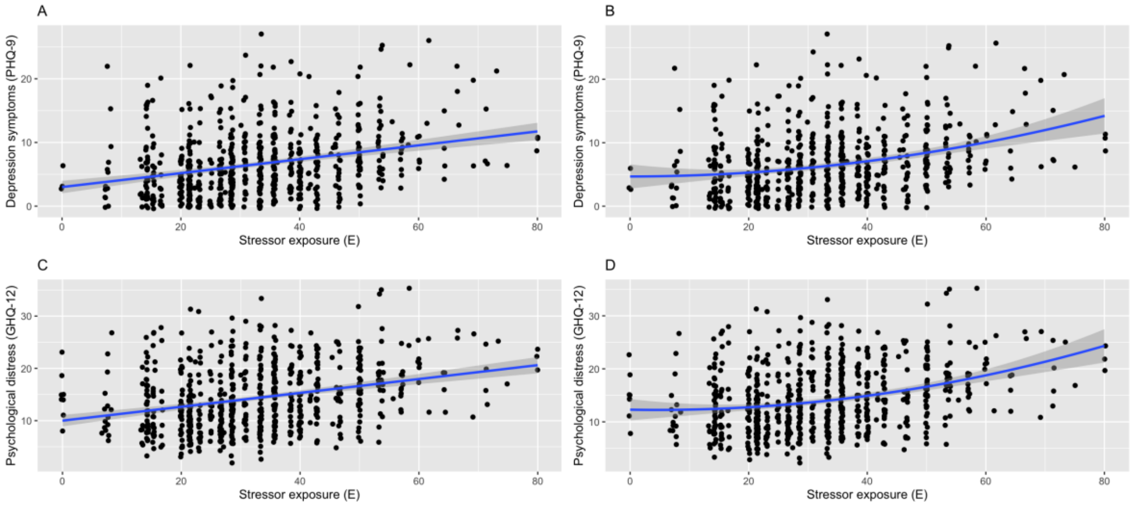

Supplementary Figure 1

Association between stressor exposure (E) and mental health outcomes (P) across waves in the complete-cases sample. The upper row shows the linear (panel A) and quadratic (panel B) fits for depression symptoms, as measured by the 9-item Patients Health Questionnaire (PHQ-9). The lower row shows the linear (panel C) and quadratic (panel D) fits for psychological distress, as measured by the 12-item General Health Questionnaire (GHQ-12). Best fitting models were used to calculate cross-sectional stressor reactivity (SR) for each outcome.

Supplementary Figure 2

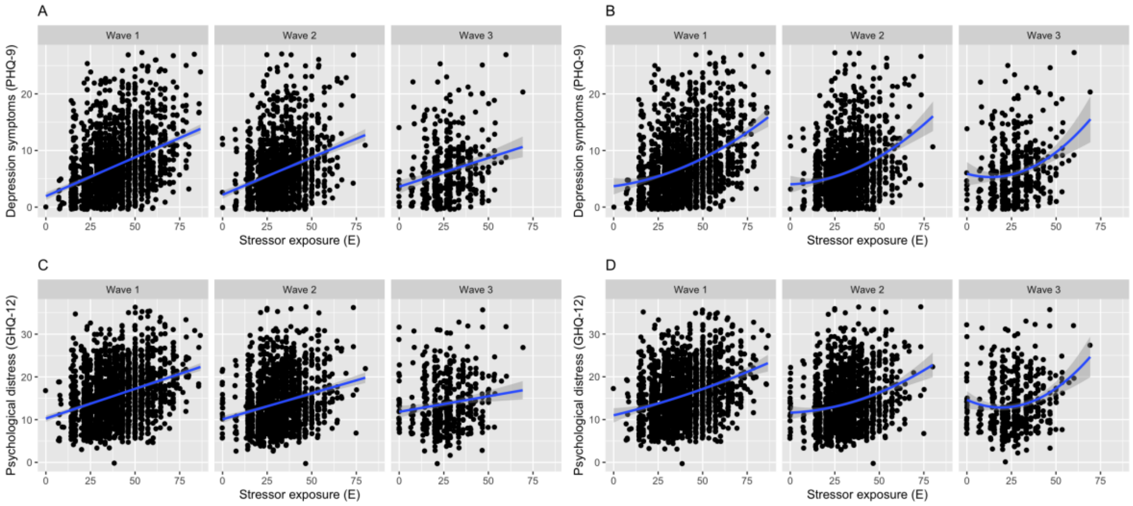

Supplementary Figure 2

Association between stressor exposure (E) and mental health outcomes (P) by wave in the full sample. The upper row shows the linear (panel A) and quadratic (panel B) fits for depression symptoms, as measured by the 9-item Patients Health Questionnaire (PHQ-9). The lower row shows the linear (panel C) and quadratic (panel D) fits for psychological distress, as measured by the 12-item General Health Questionnaire (GHQ-12). Best fitting models were used to calculate cross-sectional stressor reactivity (SR) across outcomes and waves.

Supplementary Figure 3

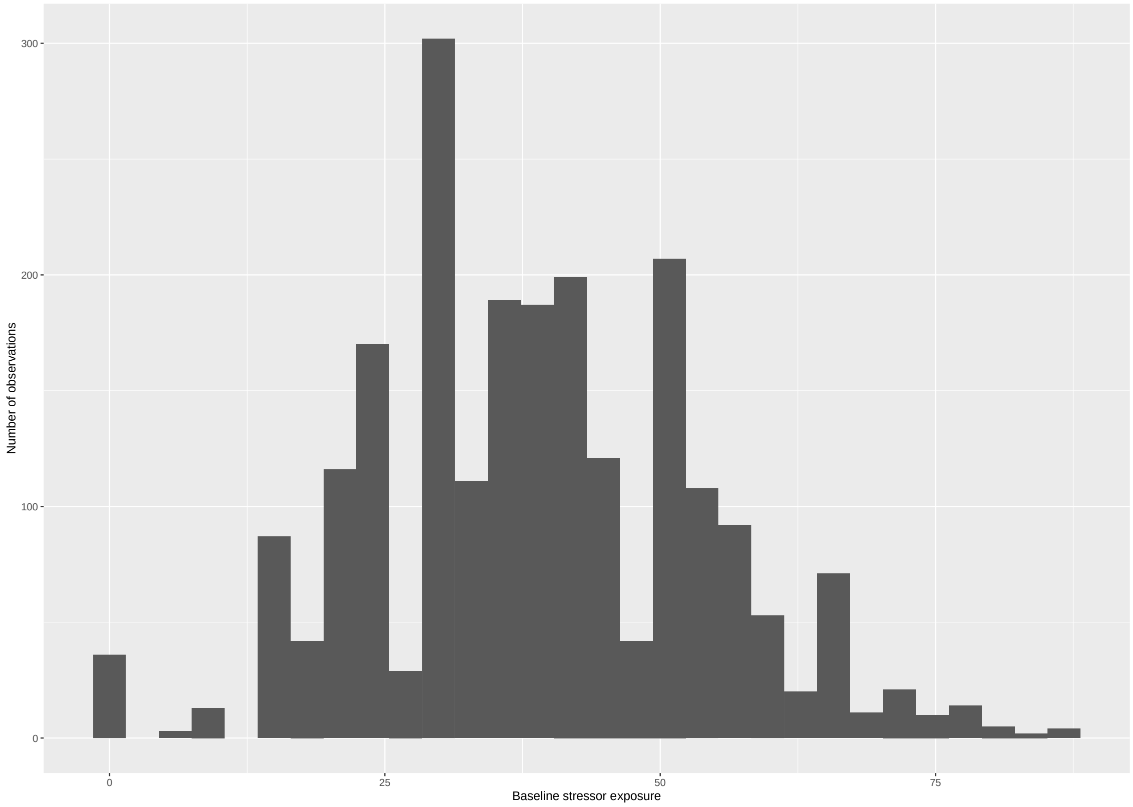

Supplementary Figure 3

Frequency of stressor exposure (E) scores at baseline in the full sample (N = 2,422). E scores range from 0 to 100 and are available for 2,265 participants (157 missing observations). The distribution is not normal, based on the Shapiro-Wilk normality test ($W = 0.99$, $p < 0.001$)

Supplementary Figure 4

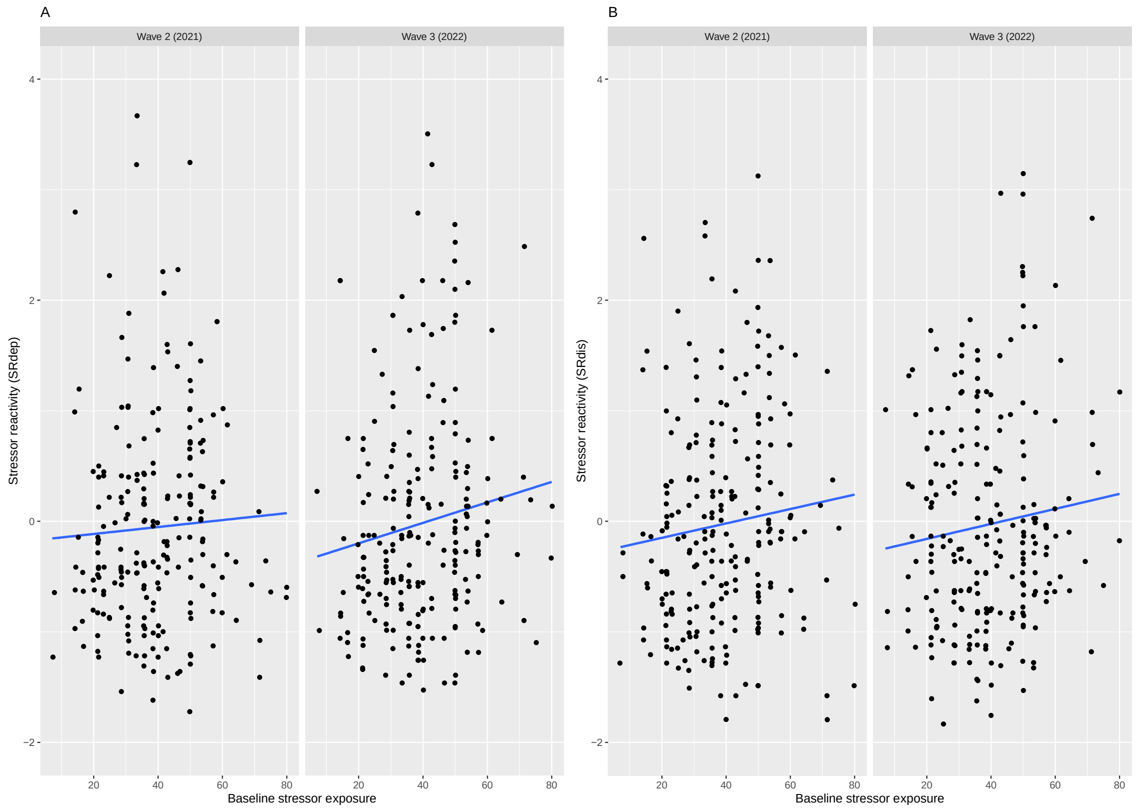

Supplementary Figure 4

Association between stressor exposure (E) at baseline and stressor reactivity (SR) scores at follow-up in the complete-cases sample. Depression SR scores (SRdep, panel A) are computed using the 9-item Patients Health Questionnaire (PHQ-9) and general distress SR scores (SRdis, panel B) are computed using the 12-item General Health Questionnaire. The effects of baseline E were not significant ($p > 0.22$).

Supplementary Table 1

#	Item (original wording)	Supporting sources
1	Have you been deployed.	Associated with poor mental health outcomes in Mediavilla et al. (2021), Moro et al. (2022)
2	Working 6 or more days a week.	Similarity to item in stressor list in Veer et al., (2021)“Increased workload”
3	Working an average of more than 12 hours per day.	Similarity to item in stressor list in Veer et al. (2021) “Increased workload” See also Moro et al. (2022)
4	Having to take triage decisions.	Associated with poor mental health outcomes in Amador-Perilla et al. (2023), Chou et al. (2022), Mediavilla et al. (2021)
5	Contact with COVID-19 patients (suspected or confirmed)	Similarity to item in stressor list in Veer et al. (2021) “Being at an increased risk for infection (e.g., at work).”
6	Working in a COVID-19 unit.	Associated with poor mental health outcomes in Czepiel et al. (2024), Lai et al. (2020), Moro et al. (2022), Paniagua-Avila et al. (2022)
7	Stigma or discrimination based on profession.	Associated with poor mental health outcomes in Kogan et al. (2023), Mediavilla et al. (2023), Moro et al. (2022), Zhang et al. (2023)
8	Experience of conflict or aggression with family members of patients.	Similarity to item in stressor list in Veer et al. (2021) “Conflicts with strangers”
9	Experience of violence due to being a HCW.	Similarity to item in stressor list in Veer et al. (2021) “Conflicts with strangers”
10	Lack of access to protective equipment.	Associated with poor mental health outcomes in Correia da Silva et al. (2022), Czepiel et al., (2022, 2024), Mediavilla et al. (2021), Paniagua-Avila et al. (2022), Umbetkulova et al. (2024).
11	Death of close contact at work due to COVID-19.	Similarity to life events list used in previous studies (Chmitorz et al., 2020) “Death of a close person”
12	Being worried about being infect with COVID-10.	Similarity to item in stressor list in Veer et al. (2021) “Being at an increased risk for infection (e.g., at work).” See also Czepiel et al. (2024), Moro et al. (2022), Paniagua-Avila et al. (2022)
13	Being worried about infecting loved ones with COVID-10.	Similarity to item in stressor list in Veer et al. (2021) “A family member, friend, or loved one is at an increased risk of a serious course of the disease in case of an infection (they belong to the so-called 'at-risk group').”
14	Testing positive for COVID-19.	Similarity to item in stressor list in Veer et al. (2021) “Having COVID-19 symptoms, or symptoms that could be related to COVID-19.”
15	Loved one diagnosed with COVID-19.	Similarity to item in Mainz Inventory of Microstressors (Chmitorz et al., 2020).
16	Death of loved one due to COVID-19.	Similarity to life events list used in previous studies (Chmitorz et al., 2020) “Death of a close person”
17	Death of patient due to COVID-19.	Similarity to life events list used in previous studies (Chmitorz et al., 2020) “Death of a close person”

Amador-Perilla, L. S., Forero-Cortés, L. D., Alvarado-Sarmiento, A., Burgos-Ortiz, L. G., Ávila-Daguer, M., & Cadena-Camargo, Y. (2023). Retos y dificultades para los tomadores de decisiones del área de la salud durante la pandemia en Bogotá: Perspectiva desde un abordaje fenomenológico interpretativo. *Revista Colombiana de Psiquiatría*. https://doi.org/10.1016/j.rcp.2023.09.002

Chou, F. L., Abramson, D., DiMaggio, C., Hoven, C. W., Susser, E., Andrews, H. F., Chihuri, S., Lang, B. H., Ryan, M., Herman, D., Susser, I., Mascayano, F., & Li, G. (2022). Factors Related to Self-Reported Distress Experienced by Physicians During Their First COVID-19 Triage Decisions. *Disaster Medicine and Public Health Preparedness*, *16*(6), 2520–2527. https://doi.org/10.1017/dmp.2021.170

Correia da Silva, A. T., Mascayano, F., Valeri, L., de Medeiros Jr, M. E., Souza, M. P. e, Ballester, D., Cavalcanti, M. T., Martínez-Alés, G., Moro, M. F., van der Ven, E., Alvarado, R., & Susser, E. (2022). COVID-19 Pandemic Factors and Depressive Symptoms Among Primary Care Workers in São Paulo, Brazil, October

and November 2020. *American Journal of Public Health*, *112*(5), 786–794. https://doi.org/10.2105/AJPH.2022.306723

Czepiel, D., Hoek, H. W., van der Markt, A., Rutten, B. P. F., Veling, W., Schirmbeck, F., Mascayano, F., Susser, E. S., & van der Ven, E. (2022). The Association Between Exposure to COVID-19 and Mental Health Outcomes Among Healthcare Workers. *Frontiers in Public Health*, *10*. https://doi.org/10.3389/fpubh.2022.896843

Czepiel, D., McCormack, C., da Silva, A. T. C., Seblova, D., Moro, M. F., Restrepo-Henao, A., Martínez, A. M., Afolabi, O., Alnasser, L., Alvarado, R., Asaoka, H., Ayinde, O., Balalian, A., Ballester, D., Barathie, J. A. L., Basagoitia, A., Basic, D., Burrone, M. S., Carta, M. G., … van der Ven, E. (2024). Inequality on the frontline: A multi-country study on gender differences in mental health among healthcare workers during the COVID-19 pandemic. *Global Mental Health (Cambridge, England)*, *11*, e34. https://doi.org/10.1017/gmh.2024.18

Kogan, C. S., Garcia-Pacheco, J. A., Rebello, T. J., Montoya, M. I., Robles, R., Khoury, B., Kulygina, M., Matsumoto, C., Huang, J., Medina-Mora, M. E., Gureje, O., Stein, D. J., Sharan, P., Gaebel, W., Kanba, S., Andrews, H. F., Roberts, M. C., Pike, K. M., Zhao, M., … Reed, G. M. (2023). Longitudinal Impact of the COVID-19 Pandemic on Stress and Occupational Well-Being of Mental Health Professionals: An International Study. *International Journal of Neuropsychopharmacology*, *26*(10), 747–760. https://doi.org/10.1093/ijnp/pyad046

Lai, J., Ma, S., Wang, Y., Cai, Z., Hu, J., Wei, N., Wu, J., Du, H., Chen, T., Li, R., Tan, H., Kang, L., Yao, L., Huang, M., Wang, H., Wang, G., Liu, Z., & Hu, S. (2020). Factors Associated With Mental Health Outcomes Among Health Care Workers Exposed to Coronavirus Disease 2019. *JAMA Network Open*, *3*(3), e203976. https://doi.org/10.1001/jamanetworkopen.2020.3976

Mediavilla, R., Fernández-Jiménez, E., Andreo, J., Morán-Sánchez, I., Muñoz-Sanjosé, A., Moreno-Küstner, B., Mascayano, F., Ayuso-Mateos, J. L., Bravo-Ortiz, M.-F., & Martínez-Alés, G. (2023). Association between perceived discrimination and mental health outcomes among health workers during the initial COVID-19 outbreak. *Spanish Journal of Psychiatry and Mental Health*, *16*(4), 221–224. https://doi.org/10.1016/j.rpsm.2021.06.001

Mediavilla, R., Fernández-Jiménez, E., Martínez-Alés, G., Moreno-Küstner, B., Martínez-Morata, I., Jaramillo, F., Morán-Sánchez, I., Minué, S., Torres-Cantero, A., Alvarado, R., Ayuso-Mateos, J. L., Mascayano, F., Susser, E., & Bravo-Ortiz, M.-F. (2021). Role of access to personal protective equipment, treatment prioritization decisions, and changes in job functions on health workers' mental health outcomes during the initial outbreak of the COVID-19 pandemic. *Journal of Affective Disorders*, *295*, 405–409. https://doi.org/10.1016/j.jad.2021.08.059

Moro, M. F., Calamandrei, G., Poli, R., Di Mattei, V., Perra, A., Kurotschka, P. K., Restrepo, A., Romano, F., La Torre, G., Preti, E., Mascayano, F., Picardi, A., Chiarotti, F., Rapisarda, V., Urban, A., Alvarado, R., Susser, E., & Carta, M. G. (2022). The Impact of the COVID-19 Pandemic on the Mental Health of Healthcare Workers in Italy: Analyzing the Role of Individual and Workplace-Level Factors in the Reopening Phase After Lockdown. *Frontiers in Psychiatry*, *13*. https://doi.org/10.3389/fpsyt.2022.867080

Paniagua-Avila, A., Ramírez, D. E., Barrera-Pérez, A., Calgua, E., Castro, C., Peralta-García, A., Mascayano, F., Susser, E., Alvarado, R., & Puac-Polanco, V. (2022). Mental Health of Guatemalan Health Care Workers During the COVID-19 Pandemic: Baseline Findings From the HEROES Cohort Study. *American Journal of Public Health*, *112*(S6), S602–S614. https://doi.org/10.2105/AJPH.2021.306648

Umbetkulova, S., Kanderzhanova, A., Foster, F., Stolyarova, V., & Cobb-Zygadlo, D. (2024). Mental Health Changes in Healthcare Workers During COVID-19 Pandemic: A Systematic Review of Longitudinal Studies. *Evaluation & the Health Professions*, *47*(1), 11–20. https://doi.org/10.1177/01632787231165076

Veer, I. M., Riepenhausen, A., Zerban, M., Wackerhagen, C., Puhlmann, L. M. C., Engen, H., Köber, G., Bögemann, S. A., Weermeijer, J., Uściłko, A., Mor, N., Marciniak, M. A., Askelund, A. D., Al-Kamel, A., Ayash, S., Barsuola, G., Bartkute-Norkuniene, V., Battaglia, S., Bobko, Y., … Kalisch, R. (2021). Psycho-social factors associated with mental resilience in the Corona lockdown. *Translational Psychiatry*, *11*(1), 67. https://doi.org/10.1038/s41398-020-01150-4

Zhang, S., Zhao, Z., Zhang, H., Zhu, Y., Xi, Z., & Xiang, K. (2023). Workplace violence against healthcare workers during the COVID-19 pandemic: A systematic review and meta-analysis. *Environmental Science and Pollution Research*, *30*(30), 74838–74852. https://doi.org/10.1007/s11356-023-27317-2

Supplementary Table 2

Changes in stressor exposure (E), mental health problems (P), as measured by the 9-item Patients Health Questionnaire (PHQ-9) and the 12-item General Health Questionnaire (GHQ-12), and stressor reactivity (SR), in the complete-cases sample (n = 332) across waves. Estimates were obtained using linear regression models.

	Time	B	95 percent CI	p-value
E	Wave 1	39.29	37.8, 40.76	0.00
	Wave 2	-7.82	-9.90, -5.74	0.00
	Wave 3	-13.99	-16.09, -11.88	0.00
PHQ-9	Wave 1	7.24	6.57, 7.92	0.00
	Wave 2	-1.01	-1.97, -0.06	0.04
	Wave 3	-1.03	-1.98, -0.07	0.04
GHQ-12	Wave 1	15.70	14.90, 16.50	0.00
	Wave 2	-1.92	-3.06, -0.78	0.00
	Wave 3	-2.29	-3.43, -1.16	0.00
SRdep	Wave 1	0.02	-0.11, 0.15	0.77
	Wave 2	-0.10	-0.28, 0.09	0.30
	Wave 3	-0.03	-0.22, 0.15	0.72
SRdis	Wave 1	0.06	-0.07, 0.19	0.36
	Wave 2	-0.17	-0.35, 0.01	0.07
	Wave 3	-0.13	-0.31, 0.05	0.16

Note.

SRdep = SR computed using PHQ-9

SRdis = SR computed using GHQ-12

Supplementary Table 3

Concurrent (wave 1) and prospective (waves 2 and 3) associations between resilience factors at baseline and stressor reactivity (SR) scores at baseline and follow-up in the complete-cases sample.

	**Wave 1 (2020)**		**Wave 2 (2021)**		**Wave 3 (2022)**	
	Unadjusted B (95% CI)	Adjusted B (95% CI)	Unadjusted B (95% CI)	Adjusted B (95% CI)	Unadjusted B (95% CI)	Adjusted B (95% CI)
SRdep						
Support from colleagues	-0.19 (-0.36, -0.02)	-0.18 (-0.35, -0.01)	-0.24 (-0.4, -0.09)	-0.24 (-0.4, -0.08)	-0.21 (-0.38, -0.03)	-0.2 (-0.38, -0.03)
Trust in the workplace	-0.05 (-0.17, 0.06)	-0.04 (-0.15, 0.07)	-0.1 (-0.2, 0)	-0.08 (-0.19, 0.02)	-0.06 (-0.18, 0.06)	-0.07 (-0.19, 0.05)
REC	-0.62 (-0.76, -0.47)	-0.59 (-0.73, -0.44)	-0.43 (-0.58, -0.29)	-0.41 (-0.56, -0.27)	-0.39 (-0.56, -0.23)	-0.39 (-0.56, -0.22)
SRdis						
Support from colleagues	-0.37 (-0.53, -0.22)	-0.37 (-0.52, -0.21)	-0.22 (-0.39, -0.06)	-0.23 (-0.39, -0.07)	-0.26 (-0.43, -0.09)	-0.26 (-0.43, -0.08)
Trust in the workplace	-0.15 (-0.26, -0.05)	-0.13 (-0.23, -0.02)	-0.09 (-0.2, 0.01)	-0.07 (-0.18, 0.04)	-0.14 (-0.26, -0.02)	-0.16 (-0.28, -0.04)
REC	-0.52 (-0.66, -0.38)	-0.51 (-0.65, -0.37)	-0.29 (-0.45, -0.14)	-0.28 (-0.43, -0.12)	-0.3 (-0.47, -0.13)	-0.32 (-0.49, -0.15)

Note. Estimates of B coefficients in the adjusted models were corrected for age and gender. REC = perceived ability to recover from stress. SRdep = SR score computed using depression symptoms as measured by the 9-item Patient Health Questionnaire (PHQ-9). SRdis = SR score computed using psychological distress symptoms as measured by the 12-item General Health Questionnaire (GHQ-12)

Supplementary Table 4

Correlations between resilience factors and stressor exposure at baseline in the full sample

	Social support	Institutional trust	REC
Institutional trust	0.22 (0.18, 0.26)<br>n = 1,872		
REC	0.14 (0.10, 0.19)<br>n = 1,846	0.12 (0.08, 0.17)<br>n = 1,847	
E at baseline	-0.05 (-0.10, -0.01)<br>n = 1,873	-0.12 (-0.16, -0.08)<br>n = 2,140	-0.14 (-0.19, -0.10)<br>n = 1,848

All p-values < .01, except for E at baseline with social support (p = 0.02)

REC = Perceived ability to recover from stress

Supplementary Table 5

	Dependent variable:					
	SRdep			SRdis		
	Support from colleagues	Trust in the workplace	REC	Support from colleagues	Trust in the workplace	REC
Support from colleagues	-0.098			-0.222^{***}		
	(0.075)			(0.075)		
Trust in the workplace		-0.015			-0.041	
		(0.053)			(0.052)	
REC			-0.542^{***}			-0.668^{***}
			(0.069)			(0.069)
E at baseline	0.012^{**}	0.008^{***}	-0.003	0.003	0.007^{**}	-0.014^{***}
	(0.006)	(0.003)	(0.005)	(0.006)	(0.003)	(0.005)
Support from colleagues:E at baseline	-0.003^{*}			-0.0004		
	(0.002)			(0.002)		
Trust in the workplace:E at baseline		-0.003^{**}			-0.003^{**}	
		(0.001)			(0.001)	
REC:E at baseline			0.0004			0.004^{**}
			(0.002)			(0.002)
Constant	0.253	-0.049	1.769^{***}	0.669^{***}	0.039	2.207^{***}
	(0.249)	(0.127)	(0.227)	(0.248)	(0.126)	(0.229)
Observations	2,461	2,896	2,831	2,475	2,953	2,830
R^2	0.029	0.018	0.148	0.031	0.024	0.140
Adjusted R^2	0.027	0.017	0.147	0.030	0.023	0.139
Residual Std. Error	0.984 (df = 2457)	0.985 (df = 2892)	0.919 (df = 2827)	0.984 (df = 2471)	0.982 (df = 2949)	0.928 (df = 2826)
F Statistic	24.087^{***} (df = 3; 2457)	17.405^{***} (df = 3; 2892)	163.696^{***} (df = 3; 2827)	26.732^{***} (df = 3; 2471)	24.444^{***} (df = 3; 2949)	152.794^{***} (df = 3; 2826)
Note:	*p<0.1;* ***p<0.05;*** p<0.01					
